# Chimeric antigen receptor-based natural killer cell immunotherapy in cancer: from bench to bedside

**DOI:** 10.1038/s41419-024-06438-7

**Published:** 2024-01-15

**Authors:** Beibei Zhang, Mengzhe Yang, Weiming Zhang, Ning Liu, Daogang Wang, Liangfang Jing, Ning Xu, Na Yang, Tao Ren

**Affiliations:** 1 Institute of Biomedical Research, Yunnan University, Kunming, 650500 China; 2 Graduate School of Capital Medical University, Beijing, 100069 China; 3 Department of Oncology, Wuming Hospital of Guangxi Medical University, Nanning, 530199 China; 4 Department of Hematology, Huazhong University of Science and Technology Union Shenzhen Hospital, Shenzhen, 518052 China; 5 Department of Gastroenterology, The First Affiliated Hospital of Guangxi University of Chinese Medicine, Nanning, 530000 China; 6 Department of Neonatology, Women and Children’s Hospital of Guangxi Zhuang Autonomous Region, Nanning, 530005 China; 7 Department of Clinical Medicine, The Fifth Affiliated Hospital of Guangxi Medical University, Nanning, 530000 China; 8 Department of Ultrasound, The Second Affiliated Hospital of Kunming Medical University, Yunnan, 650101 China; 9 Department of Oncology, The Fifth Affiliated Hospital of Guangxi Medical University, Nanning, 530000 China

**Keywords:** Cancer therapy, Cancer therapy

## Abstract

Immunotherapy has rapidly evolved in the past decades in the battle against cancer. Chimeric antigen receptor (CAR)-engineered T cells have demonstrated significant success in certain hematologic malignancies, although they still face certain limitations, including high costs and toxic effects. Natural killer cells (NK cells), as a vital component of the immune system, serve as the “first responders” in the context of cancer development. In this literature review, we provide an updated understanding of NK cell development, functions, and their applications in disease therapy. Furthermore, we explore the rationale for utilizing engineered NK cell therapies, such as CAR-NK cells, and discuss the differences between CAR-T and CAR-NK cells. We also provide insights into the key elements and strategies involved in CAR design for engineered NK cells. In addition, we highlight the challenges currently encountered and discuss the future directions in NK cell research and utilization, including pre-clinical investigations and ongoing clinical trials. Based on the outstanding antitumor potential of NK cells, it is highly likely that they will lead to groundbreaking advancements in cancer treatment in the future.

## Facts

- As a crucial immune cell type, natural killer (NK) cells function as the “first responders” in the context of cancer development.
- With the rapid advancement of immunotherapy, particularly in cell therapy, research on NK cells has intensified due to their pivotal role in disease treatment.
- An increasing number of NK cell-based therapeutics are under development, holding substantial promise for future breakthroughs in cancer treatment.
- Discussing the biological background, cell sources, and the use of NK cells in disease therapy, as well as the primary advantages and disadvantages of engineered NK cells (e.g., chimeric antigen receptor [CAR]-NK cells), is a valuable avenue for future research in cell therapy.

## Open questions

- What is the current understanding of NK cell development, function, and their application in disease therapy?
- How do NK cells exert their anticancer effects, and what are the associated mechanisms?
- What is the rationale behind CAR cell therapy, and how do CAR-T and CAR-NK cells differ? What elements and strategies are relevant in the CAR design for engineered NK cells?
- What are the existing challenges and development directions for this novel technology of CAR-NK cells? Furthermore, what is the current status of CAR cell therapy from laboratory research to clinical application (bench to bedside)?

## Introduction

Cancer, a significant class of diseases, ranks among the leading causes of human mortality. This malignant disease now stands as the primary cause of death, second only to cardiovascular and cerebrovascular diseases [1]. In addition to traditional treatment methods such as surgery, chemotherapy, and radiotherapy [2], current cancer therapies have rapidly evolved, including targeted therapy and immunotherapy [3]. Targeted therapy is based on the advancements of cellular and molecular biology, and it seeks to inhibit cancer cells by identifying specific target molecules expressed by these cells. While notable progress has been made in enhancing therapeutic effects and reducing side effects, challenges such as drug resistance and off-target effects continue to persist in targeted therapies. On the other hand, immunotherapy has rapidly developed in recent years. It is primarily divided into two categories: immune checkpoint blockade therapy [4] and immune cell therapy [5]. These approaches have yielded promising clinical recoveries for certain patients, indicating a bright future for cancer treatment.

Natural killer (NK) cells, as a vital type of immune cell, possess dual functions involving cytotoxicity and immune regulation [6]. These cells originate from hematopoietic stem cells (HSCs) in the bone marrow and acquire the ability to differentiate between “friend” and “foe” through self-major histocompatibility complex (MHC) I molecule recognition [7]. NK cells exert broad-spectrum anticancer effects without the need for specificity and MHC restriction typically observed in cancer cell inhibition. They act as the “first responders” in the battle of the immune system against cancer development and viral infections. NK cells can spontaneously identify aberrant cells in the body, swiftly eliminating them via cytotoxic means while simultaneously generating various pro-inflammatory cytokines and chemokines. Furthermore, they have the capacity to activate other immune cells, initiating the adaptive immune response [8]. NK cells were initially discovered in the 1970s and primarily play a role in combating infected microorganisms and malignantly transformed allogeneic and autologous cells. As a distinct population of innate lymphocytes, NK cells inherently possess the ability to recognize and eliminate virus-infected and cancer cells. This recognition and elimination capacity rendered NK cells safe and effective as immunotherapeutic agents for patients with advanced leukemia nearly two decades ago [9]. In recent years, NK cells have received increasing attention as potential candidates for immunotherapy.

With the rapid development of immunotherapy, especially in the realm of cell therapy, research on NK cells has become increasingly intensive due to their pivotal role in disease treatment. In this paper, we aim to elucidate the biological background, cell sources, and the use of NK cells in disease therapy. We have also discussed the principal advantages and disadvantages of engineered NK cells, offering valuable insights into future avenues for NK cell therapy research.

## NK cell biology

### NK cell origination, development and classification

Peripheral blood mononuclear cells (PBMCs) stem from HSCs in the bone marrow, responsible for generating all the blood cells in the immune system through hematopoiesis [10]. HSCs possess the capacity to differentiate into two primary lineages: myeloid (including monocytes, macrophages, granulocytes, megakaryocytes, dendritic cells, and erythrocytes) and lymphoid (involving T cells, B cells, and NK cells). In healthy human bodies, approximately 70–90% of PBMCs are lymphocytes, and the majority of T cells remain in a naive state (mature but not stimulated by antigens). Only a small fraction of T cells become activated upon antigen recognition, initiating a cell-mediated immune response. Similarly, in normal human bodies, B cells predominantly exist in a naive or memory state, awaiting antigen stimulation and constituting approximately 5–15% of lymphocytes. Upon activation, B cells differentiate into plasma cells, which release substantial quantities of antibodies, triggering a humoral immune response. NK cells account for approximately 5–10% of the lymphocyte population and play a crucial role in the body’s innate immunity [11].

**Fig. 1:**
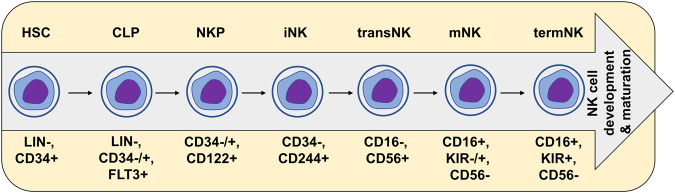
The maturation steps of NK cells in hematopoietic stem cell (HSC) differentiation and hematopoiesis process.

The development of NK cells primarily occurs from common lymphoid progenitors (CLPs) in the bone marrow, progressing through distinct stages, mainly including NK precursors (NKP), immature NK cells, and mature NK cells as illustrated in Fig. 1. In the course of normal hematopoiesis, one of the earliest markers of NK cells is IL2RB (CD122), which is expressed when CD34^+^ CLPs enter the NK cell fate lineage. Recent research has revealed the complexity of NK cell development. A “branched” model associated with NK cell development has been described, and the investigation into NK cell trafficking, tissue residence, and tissue-specific specialization is ongoing [12–16]. Subpopulations of NK cell precursors can be differentiated by their varied gene expression, including markers such as CD34, KIT, KLRB1, CD244, and IL-15R [17]. To identify immature NK cells, molecular markers like CD244, natural toxicity receptor (NCR)1, NCR2, NCR3, and KLRB1 have been reported. Subsequent differentiation to the mature stage is characterized by the expression of markers such as KLRD1, ITGB2, killer Ig-like receptors (KIRs), PRF1, IFNG, CD56, and CD16 [18]. The CD56^bright^ subset is often considered an early stage of NK cell maturation [19]. Notably, CD56^bright^ cells exhibit a higher proliferative capacity than CD56^dim^ cells, likely due to their prominent expression of several key proteins, including CCR7, CSF2, CXCR3, IL2RB, KLRC1, and SELL [20]. This subpopulation is known for its elevated IFNG expression. Downregulation of CD56 is followed by CD16 expression, resulting in the CD56^dim^ NK cell subset [21]. This subgroup is distinguished by its potent cytotoxic activity [22, 23], with KIRs being among the genes associated with this subgroup [24]. Additional genes linked to the CD56^dim^ subset include CX3CR1 [25], KLGR1 [26], and PRF1 [12].

While the CD56^bright^ cells make up a smaller portion, estimated at only 5–10% of the total NK cell population in peripheral blood (PB), the CD56^dim^ NK cells constitute over 90%. However, CD56^bright^ cells are more prevalent in specific tissues, including secondary lymphoid tissues [11, 21]. Recent findings have suggested that NK cell development can also take place in secondary lymphoid tissues such as lymph nodes and the spleen [27]. During this developmental phase, NK cells are trained to recognize MHC-I, thereby avoiding the targeting of healthy, normal cells.

### NK cell membrane protein characteristics and biology functions

**Fig. 2:**
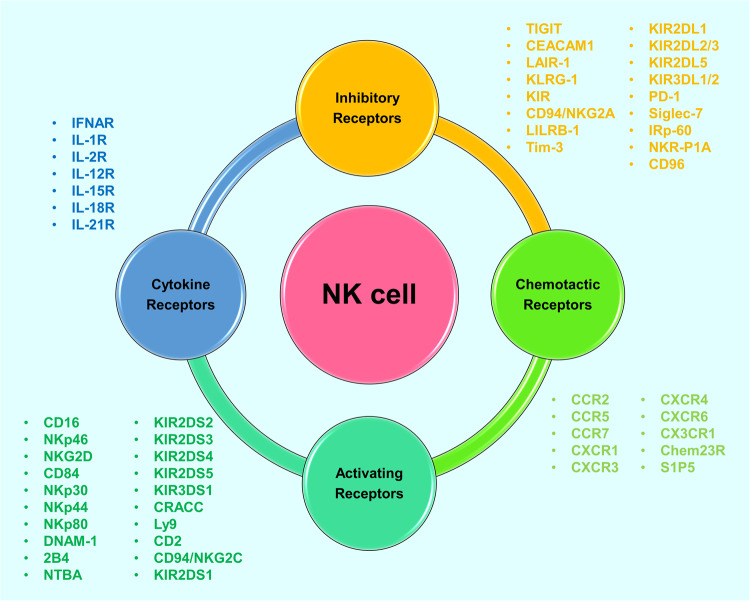
Categories of cell surface proteins expressed on NK cells. NK cells primarily express different cell surface proteins exhibiting various effects.

**Fig. 3:**
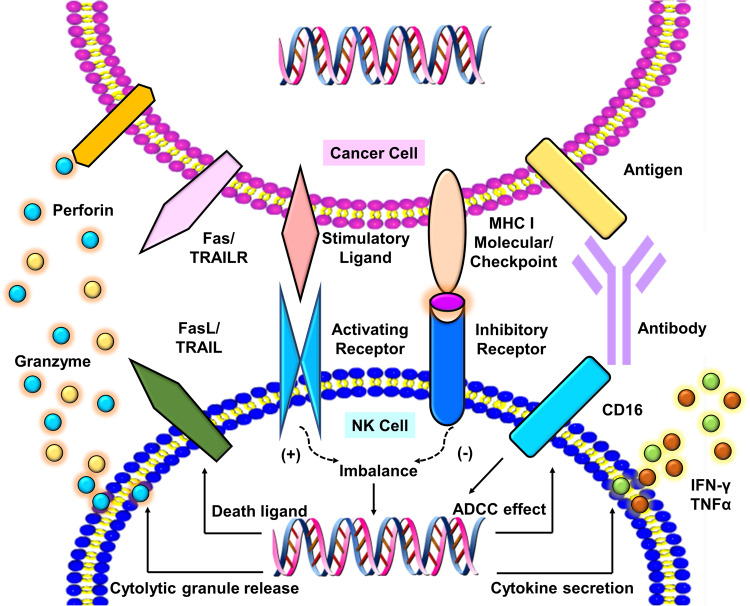
NK cells exhibit anticancer effects through diverse mechanisms. NK cells exhibit broad-spectrum anticancer effects via various mechanisms, including antibody-dependent cell-mediated cytotoxicity (ADCC) via CD16 binding, secretion of cytokines (IFN-γ, TNFα), release of cytolytic granules (e.g., granzyme B), and expression of death ligands. The balance of NK cell activity is regulated by activating and inhibitory receptors.

NK cells eliminate infected and cancerous cells upon activation of activating receptors, such as the NCRs, which include NKp30, NKp44, and NKp46 [28–30]. As mentioned earlier, CD56^dim^ NK cells primarily display robust cytotoxic activity, whereas CD56^bright^ NK cells primarily secrete cytokines and exhibit lower cytotoxic activity. The regulation of NK cell cytotoxicity involves a complex interplay of activation and inhibition signals. The killing efficiency of NK cells on target cells relies on the delicate balance between inhibitory and activation signals, closely tied to cell membrane receptors and proteins (Fig. 2). Activated NK cells employ various mechanisms for target cell elimination (Fig. 3), including ① release of perforin and granzyme, leading to target cell lysis or apoptosis; ② promotion of FasL expression, inducing cell apoptosis; ③ release of tumor necrosis factor (TNF)-α, interferon (IFN)-γ, granulocyte macrophage colony-stimulating factor (GM-CSF), and chemokines (such as CCL1, CCL2, CCL3, CCL4, CCL5, and CXCL8) to recruit and activate other effector immune cells; ④ antibody-dependent cell-mediated cytotoxicity (ADCC). NK cells express various membrane proteins, as depicted in Fig. 2, to modulate inhibitory and activation signals [16]; however, they lack T-cell receptors (TCRs) and B-cell receptors, among which CD56 [31] (an adhesion molecule mediating homotypic adhesion) and CD16 [32] (a low-affinity Fc receptor FcγRIII, contributing to NK cell-mediated ADCC) serve as primary surface markers. Unlike T cells, NK cells do not express antigen-specific recognition receptors. The surface receptors of NK cells are broadly categorized into activating and inhibitory receptors, both capable of recognizing classical or non-classical human MHC-class I molecules on normal cells. The interaction between MHC-I molecules and inhibitory receptors signals convey a “Do not eat me” message to NK cells, enabling cell evasion from NK cell-mediated killing [33].

## NK cell engineering

**Fig. 4:**
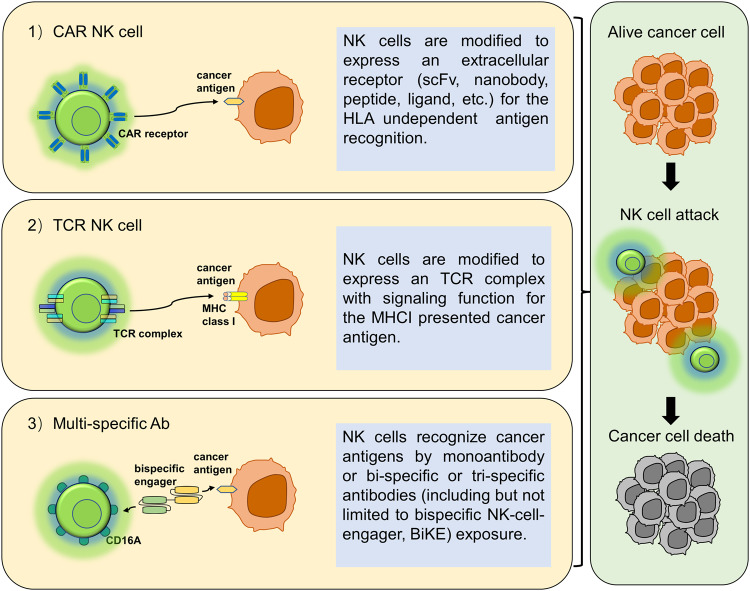
Approaches in NK cell engineering against cancer. **1**) CAR-NK cells: NK cells that are modified to express extracellular receptors (such as scFvs, nanobodies, peptides, and ligands) for HLA-independent antigen recognition. **2**) TCR NK cells: NK cells that are modified to express TCR complexes for MHC-I presented cancer antigens. **3**) Multi-specific Ab-based NK cell therapy: NK cells that utilize mono-antibodies, bi-specific antibodies, or tri-specific antibodies to recognize cancer antigens (including but not limited to bi-specific NK-cell-engager, BiKE).

Numerous studies suggest the presence of potential neoantigens, such as HSP70, expressed exclusively on tumor cells and absent in normal cells, can activate NK cells. This discovery opens a promising avenue in target-driven cell-based immunotherapies, as the HSP70 protein becomes a viable target for NK cells [34–36]. Currently, several approaches exist in NK cell engineering (Fig. 4). A prominent method involves chimeric antigen receptors (CARs), receptor proteins that confer immune cells with the ability to target specific antigenic proteins. CAR-T cell therapy has achieved significant success in hematological malignancies, including acute lymphoblastic leukemia [37, 38] and diffuse large B-cell lymphoma [39]. Various CAR-T therapies have gained approval [40–42]. While CAR-T cell therapy stands as a groundbreaking biotechnological advancement in cancer treatment, it faces challenges such as side effects, toxicity [43], T cell exhaustion [44], and limited efficacy against solid tumors [45]. Presently, new cell therapies such as CAR-NK, CAR-NKT, CAR-macrophage (CAR-M), CAR-Treg, CAR-γδT, with CAR technology at their core, have emerged, especially CAR-NK showing promising prospects in tumor immunotherapy [46].

### NK cell sources

**Fig. 5:**
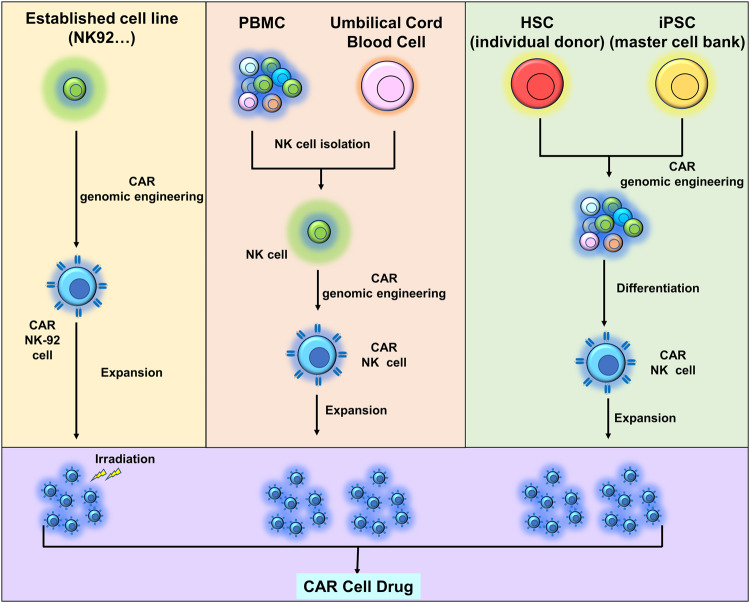
Four primary sources of NK cells in NK cell engineering. There are four primary sources of NK cells including peripheral blood (PB), induced pluripotent stem cells (iPSC), mesenchymal stem cells (e.g., umbilical cord blood), and NK cell lines (e.g., NK-92), especially in CAR-NK cell therapy.

There are four primary sources of NK cells in NK cell engineering (e.g., CAR-NK), depicted in Fig. 5: Peripheral blood (PB), induced pluripotent stem cells (iPSCs), mesenchymal stem cells (e.g., umbilical cord blood), and NK cell lines (such as NK-92, etc.). As previously mentioned, NK cells, as a subset of lymphocytes, possess the ability to recognize and eliminate tumor cells without prior sensitization, antibody involvement, and are not restricted by MHC. Commonly utilized markers for NK cells in experiments include CD16 and CD56. The characteristics and limitations of NK cells from different sources in clinical treatment are detailed below.

#### Peripheral blood (PB-NK)

NK cells derived from the patient’s own body or a healthy donor. Due to disease and treatment limitations, the function of the patient’s own NK cells may be compromised. Allogeneic NK cells are clinically favored [47], but careful T cell removal is essential to mitigate graft-versus-host disease (GVHD) [48]. PB-NK cells are mature and do not necessitate induction of differentiation, but gene transduction efficiency is relatively low. Prolonged in vitro expansion may result in shortened telomeres and reduced cytotoxicity. Cryopreservation diminishes the viability and toxicity of PB-NK cells. NK cell therapy typically involves reinfusion of 10^6^–10^8^ cells per kilogram of body weight [49, 50], yet the proportion of NK cells in PB is low [51], posing challenges for large-scale in vitro culture. However, there are advantages; for instance, these cells are mature and bypass the need for an extended differentiation period.

#### Umbilical cord blood (UCB-NK)

These cells exhibit high proliferation efficiency, allowing real-time selection of human leukocyte antigen (HLA)-mismatched products by establishing an NK cell bank [52]. Cord blood serves as a promising source for NK cells in clinical applications, with two main strategies: direct use of NK cells in cord blood or differentiation of HSCs in cord blood into NK cells. It is crucial to note that mesenchymal cells in cord blood are not utilized for these purposes. Cord blood remains a relatively stable source of NK cells. Due to their high proliferative capacity, only 10% of a cord blood unit is required to generate a nearly pure cell pool of over 10^9^ NK cells within 2 weeks, typically suitable for one treatment cycle [53, 54]. However, UCB-NK cells are not fully differentiated, exhibiting relatively low expression of NK receptors and limited cell inhibition ability, with a potential risk of tumorigenesis in allogeneic transplantation [55]. Nevertheless, they demonstrate a robust bone marrow homing ability [56]. Moreover, the higher proportion of hemoglobin and red blood cells in cord blood can impact the isolation and culture of PBMCs.

#### Stem cell-derived NK cells

NK cells are commonly induced from human embryonic stem cells (hESCs), HSCs or iPSCs [57], with an expansion period extending beyond 3–5 weeks. This extended period helps mitigate the heterogeneity of NK cells between the recipient and the donor. However, NK cells induced by iPSCs present potential risks of malignant transformation and tumorigenesis in vivo, along with the possibility of triggering unexpected immune responses due to their potential immunogenicity. iPSCs efficiently clone, expand, and differentiate in vitro, producing a substantial quantity of uniform NK cell products [58]. Nevertheless, iPSC-derived NK cells often express low levels of endogenous CD16, a drawback that can be addressed through genetic engineering [59, 60]. Moreover, iPSCs may retain DNA methylation signatures consistent with their somatic tissue origin, contributing to “epigenetic memory,” which could influence the development of specific cell lineages distinct from the donor cells. Systemic administration of cytokines in a clinical setting is highly undesirable due to its expense and potential dangers. Additionally, iPSC-derived cells carry the risk of malignant transformation and potential immunogenicity, leading to the destruction of ES cells and even adverse immune responses such as cytokine release storms [61].

#### NK cell line

Various NK cell lines, including NK-92, HANK-1, KHYG-1, NK-YS, NKG, NK101, NK3.3, YTS, and NKL, have been constructed to date, serving as excellent cell models for studying NK cell biology and associated applications. Among these, the NK-92 cell line is the only one applied in clinical trials, demonstrating a relatively satisfactory response outcome with controllable adverse effects. NK-92 cells lack the CD16 receptor-mediated ADCC effect, but this can be addressed through modification [62]. These cells are easily genetically manipulated, allowing effective introduction of exogenous genes through electroporation without the need for viral vectors [63]. Because NK-92 is a tumor-derived aneuploid immortalized cell line [64], it requires irradiation before use to inhibit in vivo proliferation [65], negatively impacting long-term persistence and overall therapeutic potential.

### CAR-NK cells

CAR-NK cells typically share the similar CAR structures as CAR-T cells. NK cells enhance their cytotoxic capacity and cytokine production through co-stimulatory molecules like NKG2D and CD244, providing probably stronger tumor-specific targeting and cytotoxicity than CAR-T cells [66]. CAR-NK cell therapy is a potential alternative to CAR-T therapy due to several unique features. Firstly, allogeneic NK cells are generally safe for adoptive cell therapy (ACT) as they do not typically mediate GVHD [66]. Moreover, NK cells only secrete small amounts of IFN-γ and GM-CSF [67], without producing IL-1 and IL-6 that initiate cytokine release syndrome (CRS) [68]. Secondly, in addition to inhibiting cancer cells through single-chain antibody recognition of tumor surface antigens, NK cells can also recognize various ligands through multiple receptors such as NCRs (NKp46, NKp44, and NKp30), NKG2D, and DNAM-1 (CD226) [69–71]. Lastly, NK cells are abundant in clinical samples [13] and can be generated from various sources, including PB [72], UCB [54], hESCs, iPSCs [57], and even NK-92 cell lines [73] as mentioned above.

**Fig. 6:**
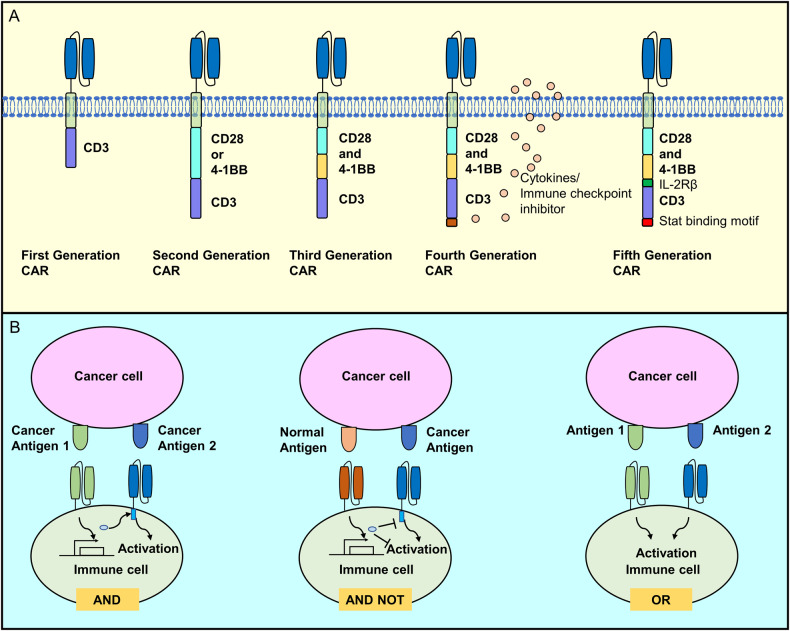
Generations of CARs and logic-gated control of CAR cells. **A** The first generation of CAR contained only CD3ζ. The second and third generations added one or two co-stimulatory domains, respectively, building on the first generation. The fourth generation introduced a cytokine secretion segment based on the third generation, and the fifth generation incorporated special binding motifs. **B** Logic-gated control of CAR cells was developed for precision therapy and to avoid potential toxicity, including “AND,” “AND NOT,” and “OR” in three logic-gated control manners.

Similar to CAR-T cells, the functional CAR molecule expressed on NK cells comprises three components: an extracellular domain, a transmembrane region, and an intracellular signaling domain (Fig. 6A). The extracellular domain includes a signal peptide and a single-chain antibody fragment (scFv) responsible for recognizing the antigen. A hinge region connects this structure to the transmembrane region, which, in turn, links to the intracellular domain containing the activation signal. The commonly utilized transmembrane segment for CAR-NK is adapted from CD3ζ, CD8, or CD28, with T cell-specific CD8 and CD28 being the most frequently employed [74]. The intracellular segment is pivotal for cell activation post-reception of the target antigen signal and constitutes a linear structure of co-stimulatory molecules and signaling domains recruited downstream of signal transduction. Successful CAR design is achieved via a combination of meticulous design and functional testing. The evolution of CAR generations includes the first generation containing only CD3ζ [75], the second and third generations adding one or two co-stimulatory domains, respectively, based on the first generation [76], the fourth generation incorporating a cytokine secretion segment based on the third generation [77], and the fifth generation introducing a special binding motif (Fig. 6A). Logic-gated control of CAR cells has also been developed to achieve precision therapy and avert potential toxicity (Fig. 6B). Currently, the second-generation CAR structures CD28-CD3ζ and 41BB-CD3ζ are most commonly used in the field of CAR-NK, whereas in the third generation, CD28-41BB-CD3ζ is also frequently employed [74, 78]. A detailed description of each CAR element is discussed below.

#### Vector backbone and promoter

The vector backbone incorporates all elements necessary for CAR expression, including a promoter, polyA signal, and transcriptional regulatory fragments. The choice of promoter directly impacts the expression of the transgene. Current reports on CAR-NK cells reveal the use of various promoters to drive CAR expression, whether derived from cell lines [79] or primary NK cells [80]. In primary CAR-NK and CAR-NK cell lines, viral promoters (such as MPSV and MMLV) are more commonly utilized than constitutively active promoters (such as EF1α and PGK) [81].

#### Signal peptide

Signal peptides exhibit substantial heterogeneity, leading to varying levels of protein production efficiency. For CAR-NK and CAR-T cells, comparative studies identifying the optimal signal peptide are lacking. Currently, CD8a-SP is the most commonly used signal peptide sequence for NK cells, and immunoglobulin heavy or light chain signal peptides are reported for NK cell lines [82].

#### Single-chain antibody fragment (scFv)

The scFv serves as the tumor antigen-binding domain of CAR [83], determining the specificity and function of CAR-NK cells. As single-chain antibodies deviate from the natural form of antibodies, the order of the heavy and light chains is artificially determined [84]. For CAR-NK designs, the VH-VL direction is preferred over the VL-VH direction [85–87]. Fujiwara et al. revealed that the order of the heavy and light chains does not affect the expression of CARs on T cells [88].

Furthermore, cells can be equipped with multiple scFvs, thereby expanding the antigen recognition capacity of CAR effector cells. Several options exist: the CAR can be transduced with a two-element vector, inducing the expression of two CAR constructs; or two scFvs can be fused in one construct, creating a “single-handle” CAR with tandem scFvs [44, 89]. While these technologies have been utilized to produce CAR-T cells [90], their application in CAR-NK cells is not well-documented. In most current clinical CAR-T cell trials, single-chain antibodies derived from mouse antibodies are commonly used, increasing the risk of GVHD in anti-mouse IgG cells. This risk can be mitigated through humanization or screening of fully human antibodies [91]. Unfortunately, even humanized scFvs may induce host anti-idiotypic immune responses due to the chimeric nature of CAR receptors [92]. However, in the limited number of CAR-NK clinical trials to date, no major adverse effects associated with anti-CAR immune responses have been identified [66]. Moreover, several other forms like nanobody were also explored to serve as the tumor antigen-binding domain.

#### Linking region

The linking region between the heavy and light chains contributes to stabilizing the conformation of the single-chain antibody. A too-short linking region may lead to multimer formation, whereas a too-long linking region can cause hydrolysis or reduce the association between VH and VL domains [93]. For CAR-NK cells, the GGGGS pentapeptide is widely used in multimers, typically in 3 repeats. Another linker designed to enhance proteolytic stability is the Whitlow “218” linker (GSTSGSGKPGSGEGSTKG) [88, 94].

#### Hinge region

The hinge region, the extracellular domain of the CAR connecting the single-chain antibody unit and transmembrane domain, maintains the stability required for robust CAR expression and activity in effector cells. Most CAR-NK constructions use derivatives of CD8α or CD28 extracellular domains or IgG-based hinge regions. The type and length of the hinge region significantly affect the functional activity of CAR [95]. Although most information comes from CAR-T, the direct transformation into CAR-NK remains unproven. A direct comparison between CD28 and CD8α hinge regions revealed that CD28 is more likely to promote CAR molecule dimerization, resulting in a stronger activation stimulus [93]. While beneficial, this can also lead to more serious adverse effects. IgG-based hinge regions, made up of the Fc portion of IgG1 or the CH2/CH3 domains of the Fc portion, offer flexibility in structure. The length of the hinge region can be adjusted to adapt to antigen recognition; however studies have revealed that a shorter spacer region results in higher cytokine production, faster CAR cell proliferation, and improved persistence and antitumor effects in vivo [95].

#### Transmembrane domain

The transmembrane domain connects the CAR extracellular domain and the intracellular activation signal domain. The most commonly used transmembrane domains for CAR-NK originate from CD3ζ, CD8, and CD28. The choice of transmembrane domain influences the activation extent of the CAR construct in cellular functions. Transmembrane domains from molecules typically expressed on NK cells, such as DNAM-1, 2B4, and NKG2D, lead to increased CD107a degranulation and higher cytotoxicity. Thus, the specific source of the transmembrane domain determines the activity of CAR-NK [96]. An important aspect of the transmembrane domain is that it should follow the natural orientation (order of N-terminal to C-terminal) of transmembrane proteins on NK cells. At present, CD8α- and CD28-modified transmembrane regions are most common in primary CAR-NK cells, whereas CD28 is the preferred transmembrane region for CAR-NK cell lines [93].

#### Activation signal

The number of intracellular activation signals in a CAR determines its “generation”. First-generation CAR-NK cells, akin to CAR-T cells, contain only the CD3ζ signal. Second-generation and third-generation CAR-NKs carry one and two additional co-stimulatory signals, respectively, typically derived from the CD28 family (CD28 and ICOS), the TNF receptor family (4-1BB, OX40, and CD27), or the signaling lymphocytic activation molecule-related receptor family (2B4) [97]. The published CAR-NK clinical trial used a second-generation CAR-NK construct that improved activity by incorporating IL-15 expression. Most current CAR structures depend on the CD3ζ chain signaling domain, and robust activation signals are crucial for eliciting potent antitumor responses but may also result in rapid effector cell exhaustion. Combinations of co-stimulatory domains can be employed to calibrate desired immune cell responses. CD28-based CARs exhibit a faster effector profile than 4-1BB-based CARs, inducing higher levels of IFN-γ, granzyme B, and TNF-α. However, this strong co-stimulatory signal also results in activation-induced cell death (AICD). Conversely, 4-1BB-CD3ζ signaling preferentially induces memory-related genes and sustained antitumor activity [98, 99]. This difference may be attributed to the amelioration of T cell exhaustion induced by the 4-1BB domain in contrast to the CD28 domain [44]. CD3ζ was universally used as the primary activation domain in studies of CAR-NK cell lines and primary CAR-NK cells, with approximately half carrying an additional activation domain, generally with the addition of 4-1BB or CD28. For third-generation constructs, the combination CD28/4-1BB/CD3ζ is most commonly employed. Intracellular signaling domains, such as CD28, 4-1BB, and OX40, often function to trigger immune cell activation and inhibition [100]. A recent report using iPSC-derived CAR-NK therapy identified the crucial role of the NKG2D transmembrane domain and emphasized the critical role of the 2B4 co-stimulation domain [96]. Different researchers also performed similar studies using 2B4 to highlight the importance of the activation signal in immune cell therapy [101, 102]. At present, four generations of CAR structures have been developed and are available for CAR-NK research.

#### CAR transfection or transduction vector

With the advancement of gene modification technology, various methods have been employed to generate CAR-NK cells. The two primary methods include viral transduction [103] (using lentiviruses or retroviruses) and transfection of naked plasmid DNA [87], transposase DNA-mediated integration [104], and mRNA electroporation [96]. Lentiviruses can efficiently transduce both periodic and non-cyclical cells and have been widely utilized in gene therapy [105]. They have been successfully used as vectors in studies on primary CAR-NK cells and CAR-NK cell lines. Both second-generation and third-generation lentiviruses have been used in preclinical studies to generate CAR-expressing NK cell lines and primary CAR-NK cells. Retroviral vectors are also commonly used for NK cells [54, 66]. Retroviruses have been used as gene therapy vectors for decades, including CAR-NK cell lines and primary NK cells [106]. In a recent phase I clinical trial, retroviral-transduced CD19 CAR-NK cells were used to treat CD19^+^ non-Hodgkin’s lymphoma and chronic lymphocytic leukemia. The study results stated that 73% of patients responded to the treatment, with 7 of 8 patients achieving a complete response. Responses were rapid, occurring within 30 days of CAR-NK administration at all dose levels. After the 1-year follow-up, expanded CAR-NK cells remained detectable [66]. Following infusion, CAR-NK DNA copy numbers remained stable in PB for up to 1 year, indicating, for the first time, that retroviral-transduced CAR-NK cells can exhibit long-term in vivo survival. Different retroviruses types have been used to generate CAR-NK cells. The RD114 retrovirus was reported to be more efficient at transducing primary NK cells than the γ retrovirus and lentivirus [107]. Although long-term stable CAR expression in NK cells can be achieved using various retroviruses, the safety of retroviral systems remains a concern, especially when compared with the safer lentiviruses. CAR-encoding mRNA electroporation is a rapid, efficient, but short-lived method. To date, mRNA electroporation has been used in CAR-NK cell lines and primary CAR-NK cell studies. Generally, expanded or activated NK cells exhibit much higher mRNA transfection efficiency than freshly isolated NK cells [108]. Because mRNA synthesis is a good manufacturing practice (GMP)-compliant manner and electroporation can be conducted in a clean room, it is feasible to generate GMP-compliant CAR-NK cells through mRNA electroporation. However, the primary disadvantage of this approach is the short window for CAR expression: after electroporation, CAR-NK cells should be infused back into the patient within 7 days. The Sleeping Beauty transposon system has also been developed. Transposon-based systems offer important advantages over conventional methods, such as the efficient introduction of CAR transgenes at predetermined locations. Transposons are primarily introduced into NK cells via electroporation and then integrated into the host genome by transposonases [50]. Clustered regularly interspaced short palindromic repeats (CRISPR)/Cas9 is another powerful genetic modification technology that introduces the Cas9 protein into NK cells along with gRNA. This technique was initially used to disrupt the CD38 gene in primary NK cells [109], aiming to prevent NK cell cannibalism in combination with daratumumab (anti-CD38) because CD38 is expressed in NK cells, multiple myeloma [110], and acute myeloid leukemia (AML) cells [111]. CRISPR/Cas9 has also been recently used to introduce some other new genes [112]. Overall, using CRISPR/Cas9 strategy is a promising strategy to precisely delete, repair, or introduce specific genes, facilitating the generation of potent antitumor NK cells.

While we have established the superiority of a specific domain among multiple candidates based on findings from T cells expressing the CAR applied into NK cells, it is crucial to note that the superiority of CAR domains is complex and depends on factors such as the target and the interaction with other domains. Further exploration is needed to determine if the results and experiences obtained from T cells are applicable to NK cells and to anticipate optimization. Moreover, the expansion of NK cells may result in an adverse phenomenon of “suicide” or “fratricide” as mentioned above, where cells recognize receptors or ligands on the surface of other similar cells and trigger cytotoxic activity against them. The Fas/FasL axis is among the most relevant mechanisms. FasL-mediated cytotoxicity plays a crucial role in NK cell function, triggering caspase-dependent apoptosis when it binds to the receptor Fas in the target cell. Fas can also serve as a steady-state mechanism for inhibiting NK cell activity expressed by NK cells, known as AICD. NKG2D is another receptor that may lead to self-killing among NK cells, which is a natural receptor primarily comprising NK, CD8^+^T, and γδ T cell expression, displaying recognition of various stress-induced ligands. Cannibalism may also occur in CAR-NK cells due to CAR ligand/antigen recognition if certain target antigens are also expressed on these effector cells [113]. Furthermore, trogocytosis is a common phenomenon that often takes place during NK cell-mediated cancer inhibition. Trogocytosis ultimately leads to the transfer of antigens to NK cells, mediating the inhibition of NK cells by other NK cells. The knock out of target antigens in effector cells can overcome fratricide, but this method is unsuitable for antigens transferred to effector cells during trogocytosis. Low affinity of CAR to antigen or optimized CAR signaling transduction may serve as alternative approaches [114].

### Popular targets of CAR-NK research

#### Solid tumor targets

In several cancer types, programmed cell death ligand 1 (PD-L1) is upregulated in the tumor microenvironment (TME) and in immunosuppressive cells [115]. Preclinical tests have revealed that PD-L1-targeted CAR-NK cells exhibit specific antitumor effects against several in vitro tumor cell lines, and exhibit robust in vivo antitumor effects against triple-negative breast cancer [116], bladder cancer [117], and lung cancer [118]. Human epidermal growth factor receptor 2 (HER2)/erythroblastic oncogene B 2 (ERBB2) is often overexpressed in breast, gastric, esophageal, ovarian, and endometrial cancers [119]. HER2 is associated with poor survival and also expressed in most glioblastomas [120]. Extensive studies have been conducted on the application of CAR constructs targeting HER2 [121]. NKG2D is an activating NK cell receptor modulating the anticancer cytotoxic potential of NK cells by interacting with its tumor-associated overexpressed ligands [122]. NKG2D ligands include MICA, MICB, and ULBPs (ULBP1, ULBP2, ULBP3, ULBP4, ULBP5, ULBP6) [123, 124]. Altogether, the targets in solid tumor (pre-) clinical therapy are relatively fewer than hematologic malignancies, but display potentials that CAR-NK cells congregate in these tumors [50].

#### Hematologic malignancy targets

**Fig. 7:**
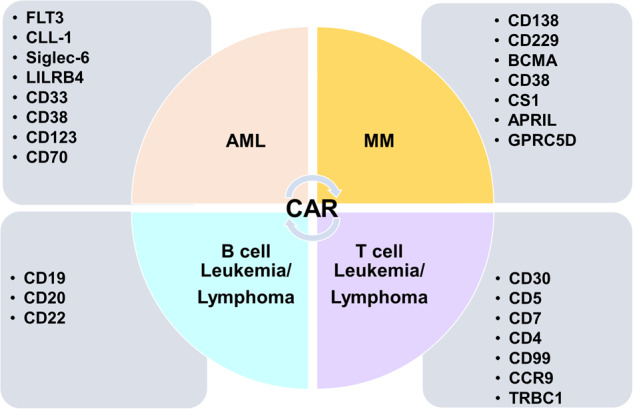
Popular targets in hematologic malignancy cell therapy. The currently popular targets in hematologic malignancy cell therapy are listed in the categories.

Clinical studies have demonstrated the significant efficacy of anti-CD19 CAR-T cell therapy in tumor immunotherapy [66]. However, CAR-T cell therapy is constrained by various adverse effects and manufacturing challenges. CAR-NK cells present themselves as an alternative therapeutic strategy for hematological malignancies, focusing on the currently popular targets (Fig. 7). Multiple myeloma (MM) is a hematologic malignancy, and numerous CAR-T and CAR-NK cell therapies are being currently developed [125], with B-cell maturation antigen being the most popular cell therapy target [126]. Furthermore, CD38 and CD138 are also common targets for MM treatment with CAR constructs [127]. CD19, CD20, and CD22 are commonly employed targets for CAR-T cell therapy in B-cell lymphoma and leukemia. Two major challenges in constructing CARs against AML include shared antigen expression and heterogeneity with hematopoietic progenitor cells. Shared antigenic expression of CD123 and CD33 can result in on-target nontumor toxicity [128, 129]. In several cases, targeting various AML-associated antigens (such as FLT3 [130], CD123 [131], CD33 [132], CLL-1 [133], and GRP78 [134]) using multiple CARs may be necessary because certain tumor-associated antigens (TAAs) may not be expressed on all leukemia cells.

### Challenges of NK cell therapy

At present, CAR-NK cells demonstrate obvious advantages compared with CAR-T cells, as summarized in previous literature [135–137] (Table 1). Registered clinical trials using these cells have seen a rapid increase, as shown in Table 2. Unlike CAR-T cell clinical applications, NK cells exhibit relatively acceptable toxicity adverse effects, avoiding effects such as cytokine release syndrome. Several clinical trial evidences support this, for instance, Tang *et al*. reported the first-in-man CD33^+^ CAR-NK-92 cell clinical trial, testing safety in patients with AML experiencing relapsed and refractory conditions. The dose of 5 × 10^9^ cells in each patient led to no evident adverse effects [138]. Additionally, Liu et al. reported phase I and II trial outcomes using CD19 CAR-NK cells, revealing a rapid response in patients with CD19^+^ cancer with relapsed or refractory conditions, without apparent associations between effectors and adverse effects, including neurotoxicity, cytokine release syndrome, or GVHD [66]. However, challenges persist in this type of immunotherapy, summarized as follows:

**Table 1:** CAR-NK cells exhibit obvious advantages compared with CAR-T cells.

Character	CAR-T	CAR-NK
Risk of GVHD	High	Low
Anti-cancer effect	High	High
Risk of Cytokine storm	High	Low
Cost of product	High	Low
Source of harvest	Limited	Wide
Potential of off-the-shelf	Low	High

**Table 2:** Representative clinical trials with CAR-NK cells.

No.	Study Title	NCT Number	Status	Conditions	Interventions
1	NKG2D CAR-NK Cell Therapy in Patients With Relapsed or Refractory Acute Myeloid Leukemia	NCT05247957	Terminated	Safety and Efficacy	CAR-NK cells
2	A Phase I/II Study of Universal Off-the-shelf NKG2D-ACE2 CAR-NK Cells for Therapy of COVID-19	NCT04324996	Unknown status	COVID-19	NK cells, IL15-NK cells, NKG2D CAR-NK cells, ACE2 CAR-NK cells, NKG2D-ACE2 CAR-NK cells
3	Allogenic CD123-CAR-NK Cells in the Treatment of Refractory/Relapsed Acute Myeloid Leukemia	NCT05574608	Recruiting	Acute Myeloid Leukemia Refractory Acute Myeloid Leukemia Recurrent	CD123-CAR-NK cells
4	The Study of Anti-CD19 CAR NK Cells in the Treatment of Relapsed/Refractory Diffuse Large B Cell Lymphoma	NCT05673447	Recruiting	Diffuse Large B Cell Lymphoma	CD19 CAR NK cells
5	Study of DLL3-CAR-NK Cells in the Treatment of Extensive Stage Small Cell Lung Cancer	NCT05507593	Recruiting	SCLC, Extensive Stage	DLL3-CAR-NK cells
6	CLDN6-CAR-NK Cell Therapy for Advanced Solid Tumors	NCT05410717	Recruiting	Stage IV Ovarian Cancer Testis Cancer, Refractory Endometrial Cancer Recurrent	Claudin6 targeting CAR-NK cells
7	Anti-CD19 CAR-Engineered NK Cells in the Treatment of Relapsed/Refractory B-cell Malignancies	NCT05410041	Recruiting	Acute Lymphocytic Leukemia Chronic Lymphocytic Leukemia Non Hodgkin Lymphoma	CAR-NK-CD19 Cells
8	Study of Anti-CD22 CAR NK Cells in Relapsed and Refractory B Cell Lymphoma	NCT03692767	Unknown status	Refractory B-Cell Lymphoma	CD22 CAR NK Cells
9	Study of Anti-CD19 CAR NK Cells in Relapsed and Refractory B Cell Lymphoma	NCT03690310	Unknown status	Refractory B-Cell Lymphoma	CD19 CAR NK Cells
10	CAR-NK Targeted CD19 for r/r B-cell Malignancies	NCT05645601	Recruiting	Adult Relapsed/Refractory B-cell Hematologic Malignancies	CD19-CAR-NK cells
11	Safety and Efficacy of Allogenic CD19-CAR-NK Cells in Treatmenting r/r B-cell Hematologic Malignancies	NCT05739227	Recruiting	Acute Lymphoblastic Leukemia B-cell Lymphoma Chronic Lymphocytic Leukemia	CD19-CAR-NK cells
12	NKG2D CAR-NK Cell Therapy in Patients With Refractory Metastatic Colorectal Cancer	NCT05213195	Recruiting	Refractory Metastatic Colorectal Cancer	NKG2D CAR-NK cells
13	Natural Killer(NK) Cell Therapy in Acute Myeloid Leukemia	NCT05987696	Not yet recruiting	AML, Adult Minimal Residual Disease	CD33/CLL1 dual CAR-NK cells Cyclophosphamid Fludarabine
14	NKG2D CAR-NK & Ovarian Cancer	NCT05776355	Recruiting	Ovarian Cancer	NKG2D CAR-NK cells
15	Anti-CD19 CAR-Engineered NK Cells in the Treatment of Relapsed/Refractory Acute Lymphoblastic Leukemia	NCT05563545	Completed	Acute Lymphoblastic Leukemia	CAR-NK-CD19 Cells
16	NKG2D CAR-NK & r/rAML	NCT05734898	Recruiting	AML	NKG2D CAR-NK cells
17	Pilot Study of NKG2D-Ligand Targeted CAR-NK Cells in Patients With Metastatic Solid Tumours	NCT03415100	Unknown status	Solid Tumours	CAR-NK cells targeting NKG2D ligands
18	Clinical Study of the Safety and Efficacy of BCMA CAR-NK	NCT05652530	Recruiting	Immunotherapy Multiple Myeloma	BCMA CAR-NK cells
19	Clinical Research of ROBO1 Specific BiCAR-NK Cells on Patients With Pancreatic Cancer	NCT03941457	Unknown status	Pancreatic Cancer	BiCAR-NK cells (ROBO1 CAR-NK cells)
20	Clinical Study of HLA Haploidentical CAR-NK Cells Targeting CD19 in the Treatment of Refractory/Relapsed B-cell NHL	NCT04887012	Recruiting	B-cell Non Hodgkin Lymphoma	CD19 CAR-NK cells
21	Clinical Study of Cord Blood-derived CAR-NK Cells Targeting CD19 in the Treatment of Refractory/Relapsed B-cell NHL	NCT05472558	Recruiting	B-cell Non Hodgkin Lymphoma	CD19 CAR-NK cells
22	Clinical Research of ROBO1 Specific CAR-NK Cells on Patients With Solid Tumors	NCT03940820	Unknown status	Solid Tumor	ROBO1 CAR-NK cells
23	Safety and Efficacy of CD123-targeted CAR-NK for Relapsed/Refractory Acute Myeloid Leukemia or Blastic Plasmacytoid Dendritic Cell Neoplasm	NCT06006403	Recruiting	Acute Myeloid Leukemia Blastic Plasmacytoid Dendritic Cell Neoplasm (BPDCN) Relapse Leukemia	CD123 targeted CAR-NK cells
24	Anti-CD19 Universal CAR-NK Cells Therapy Combined With HSCT for B Cell Hematologic Malignancies	NCT05570188	Withdrawn	B-cell Lymphoma B-cell Leukemia	CD19 UCAR-NK cells
25	CAR-pNK Cell Immunotherapy for Relapsed/Refractory CD33 + AML	NCT02944162	Unknown status	Acute Myelogenous Leukemia Acute Myeloid Leukemia Acute Myeloid Leukemia With Maturation	CD33 CAR-NK cells
26	An Exploratory Clinical Study of Anti-CD19 CAR NK Cells in the Treatment of Systemic Lupus Erythematosus	NCT06010472	Recruiting	Systemic Lupus Erythematosus (SLE)	CD19 CAR NK cells (KN5501)
27	Clinical Research of ROBO1 Specific BiCAR-NK/T Cells on Patients With Malignant Tumor	NCT03931720	Unknown status	Malignant Tumor	BiCAR-NK/T cells
28	Study of Anti-5T4 CAR-NK Cell Therapy in Advanced Solid Tumors	NCT05194709	Recruiting	Advanced Solid Tumors	5T4-CAR-NK Cells
29	Clinical Research of Adoptive BCMA CAR-NK Cells on Relapse/Refractory MM	NCT03940833	Unknown status	Multiple Myeloma	BCMA CAR-NK 92 cells
30	Study of Anti-CD19/CD22 CAR NK Cells in Relapsed and Refractory B Cell Lymphoma	NCT03824964	Unknown status	Refractory B-Cell Lymphoma	Anti-CD19/CD22 CAR NK Cells
31	PCAR-119 Bridge Immunotherapy Prior to Stem Cell Transplant in Treating Patients With CD19 Positive Leukemia and Lymphoma	NCT02892695	Unknown status	Acute Lymphocytic Leukemia Chronic Lymphocytic Leukemia Follicular Lymphoma	CD19 CAR-NK cells
32	Study of Anti-CD33/CLL1 CAR-NK in Acute Myeloid Leukemia	NCT05215015	Recruiting	Acute Myeloid Leukemia	CD33/CLL1 CAR-NK Cells
33	Single-arm, Open-label Clinical Study of SZ003 in the Treatment of Advanced Hepatocellular Carcinoma	NCT05845502	Not yet recruiting	Advanced Hepatocellular Carcinoma	SZ003 CAR-NK
34	Cord Blood Derived Anti-CD19 CAR-Engineered NK Cells for B Lymphoid Malignancies	NCT04796675	Recruiting	Acute Lymphocytic Leukemia Chronic Lymphocytic Leukemia Non Hodgkin’s Lymphoma	Fludarabine + Cyclophosphamide + CAR-NK-CD19 Cells
35	Anti-CD33 CAR NK Cells in the Treatment of Relapsed/Refractory Acute Myeloid Leukemia	NCT05008575	Recruiting	Leukemia, Myeloid, Acute	CD33 CAR NK cells Fludarabine Cytoxan
36	Anti-BCMA CAR-NK Cell Therapy for the Relapsed or Refractory Multiple Myeloma	NCT05008536	Recruiting	Multiple Myeloma, Refractory	BCMA CAR-NK Cells Fludarabine Cytoxan
37	CAR-pNK Cell Immunotherapy in CD7 Positive Leukemia and Lymphoma	NCT02742727	Unknown status	Acute Myeloid Leukemia Precursor T-Cell Lymphoblastic Leukemia-Lymphoma T-cell Prolymphocytic Leukemia	CD7 CAR-pNK cells
38	Study of TROP2 CAR Engineered IL15-transduced Cord Blood-derived NK Cells Delivered Intraperitoneally for the Management of Platinum Resistant Ovarian Cancer, Mesonephric-like Adenocarcinoma, and Pancreatic Cancer	NCT05922930	Not yet recruiting	Pancreatic Cancer Ovarian Cancer Adenocarcinoma	TROP2-CAR-NK Cyclophosphamide Fludarabine
39	NKX101, Intravenous Allogeneic CAR NK Cells, in Adults With AML or MDS	NCT04623944	Recruiting	Relapsed/Refractory AML AML, Adult MDS 1 more	NKX101 - CAR NK cell therapy
40	Umbilical & Cord Blood (CB) Derived CAR-Engineered NK Cells for B Lymphoid Malignancies	NCT03056339	Completed	B-Lymphoid Malignancies Acute Lymphocytic Leukemia Chronic Lymphocytic Leukemia	CAR NK cells Fludarabine Cyclophosphamide Mesna
41	Phase I/II Study of CAR.70- Engineered IL15-transduced Cord Blood-derived NK Cells in Conjunction With Lymphodepleting Chemotherapy for the Management of Relapse/Refractory Hematological Malignances	NCT05092451	Recruiting	B-Cell Lymphoma Myelodysplastic Syndromes (MDS) Acute Myeloid Leukemia (AML)	Cyclophosphamide CAR.70/IL15-transduced CB-NK cells Fludarabine phosphate
42	Phase I/II Study of CD5 CAR Engineered IL15-Transduced Cord Blood-Derived NK Cells in Conjunction With Lymphodepleting Chemotherapy for the Management of Relapsed/Refractory Hematological Malignances	NCT05110742	Not yet recruiting	Hematological Malignancy	Fludarabine Phosphate Cyclophosphamide CAR.5/IL15-transduced CB-NK cells
43	Immunotherapy Combination: Irradiated PD-L1 CAR-NK Cells Plus Pembrolizumab Plus N-803 for Subjects With Recurrent/Metastatic Gastric or Head and Neck Cancer	NCT04847466	Recruiting	Gastroesophageal Junction (GEJ) Cancers Advanced HNSCC	N-803 Pembrolizumab PD-L1 t-haNK
44	Induced-T Cell Like NK Cells for B Cell Malignancies	NCT04747093	Unknown status	B Cell Leukemia B Cell Lymphoma B-cell Acute Lymphoblastic Leukemia	CAR-ITNK cells
45	Clinical Study of Cord Blood-derived CAR NK Cells Targeting CD19/CD70 in Refractory/Relapsed B-cell Non-Hodgkin Lymphoma	NCT05667155	Recruiting	B-cell Non Hodgkin Lymphoma	CB dual CAR-NK19/70
46	A Study of Universal CD19-Targeted UCAR-NK Cells Combined With HSCT for B Cell Hematologic Malignancies	NCT05654038	Recruiting	B-Cell Lymphoblastic Leukemia/Lymphoma	CD19 UCAR-NK cells
47	NKX019, Intravenous Allogeneic Chimeric Antigen Receptor Natural Killer Cells (CAR NK), in Adults With B-cell Cancers	NCT05020678	Recruiting	Lymphoma, Non-Hodgkin B-cell Acute Lymphoblastic Leukemia Large B-cell Lymphoma	NKX019
48	A Study of TAK-007 in Adults With Relapsed or Refractory (r/r) B-cell Non-Hodgkin Lymphoma (NHL)	NCT05020015	Recruiting	Relapsed or Refractory (r/r) B-cell Non-Hodgkin Lymphoma (NHL)	TAK-007 Chemotherapy Agents
49	Study of Anti-Mesothelin Car NK Cells in Epithelial Ovarian Cancer	NCT03692637	Unknown status	Epithelial Ovarian Cancer	Mesothelin Car NK Cells
50	Study of Cord Blood-derived CAR NK Cells Targeting CD19/CD70 in Refractory/Relapsed B-cell Non-Hodgkin Lymphoma	NCT05842707	Recruiting	Refractory or Relapsed B-cell Non-Hodgkin Lymphoma	dualCAR-NK19/70 cell
51	CAR-pNK Cell Immunotherapy in MUC1 Positive Relapsed or Refractory Solid Tumor	NCT02839954	Unknown status	Hepatocellular Carcinoma Non-small Cell Lung Cancer Pancreatic Carcinoma	MUC1 CAR-pNK cells
52	Anti-CD19 CAR NK Cell Therapy for R/R Non-Hodgkin Lymphoma.	NCT04639739	Not yet recruiting	NHL	CD19 CAR NK
53	Universal Chimeric Antigen Receptor-modified AT19 Cells for CD19+ Relapsed/Refractory Hematological Malignancies	NCT04796688	Recruiting	Acute Lymphoblastic Leukemia Chronic Lymphoblastic Leukemia B-cell Lymphoma	Fludarabine + Cyclophosphamide + CAR-NK-CD19 Cells
54	Study of CAR.70-engineered IL15-transduced Cord Blood-derived NK Cells in Conjunction With Lymphodepleting Chemotherapy for the Management of Advanced Renal Cell Carcinoma, Mesothelioma and Osteosarcoma	NCT05703854	Recruiting	Advanced Renal Cell Carcinoma Advanced Mesothelioma Advanced Osteosarcoma	CAR.70/IL15-transduced CB-derived NK cells Fludarabine phosphate Cyclophosphamide
55	FT576 in Subjects With Multiple Myeloma	NCT05182073	Recruiting	Multiple Myeloma Myeloma	FT576 (Allogenic CAR NK cells with BCMA expression) Cyclophosphamide Fludarabine
56	Allogeneic NK T-Cells Expressing CD19 Specific CAR in B-Cell Malignancies	NCT05487651	Recruiting	NHL, Relapsed, Adult B-cell Lymphoma B-cell Leukemia	Genetic: KUR-502
57	Single-arm, Open-label Clinical Study of SZ011 in the Treatment of Ovarian Epithelial Carcinoma	NCT05856643	Not yet recruiting	Ovarian Epithelial Carcinoma	SZ011 CAR-NK
58	Single-arm, Open-label Clinical Study of SZ011 in the Treatment of Advanced Triple Negative Breast Cancer	NCT05686720	Not yet recruiting	Advanced Triple Negative Breast Cancer	SZ011 CAR-NK
59	RD13-01 for Patients With r/r CD7 + T/NK Cell Hematologic Malignancies	NCT04538599	Completed	Hematologic Malignancies	RD13-01 cell infusion
60	Gene Therapy for B-Cell Non-Hodgkin Lymphoma Using CD19 CAR Gene Transduced T Lymphocytes	NCT02134262	Unknown status	Relapsed or Refractory B-Cell Non-Hodgkin Lymphoma	Cyclophosphamide or Bendamustine Dose Level 1 Dose Level 1
61	CAR.CD19-CD28-zeta-2A-iCasp9-IL15-Transduced Cord Blood NK Cells, High-Dose Chemotherapy, and Stem Cell Transplant in Treating Participants With B-cell Lymphoma	NCT03579927	Withdrawn	CD19 Positive Mantle Cell Lymphoma Recurrent Diffuse Large B-Cell Lymphoma	Autologous Hematopoietic Stem Cell Transplantation Carmustine Cytarabine
62	Study of Anti-PSMA CAR NK Cell (TABP EIC) in Metastatic Castration-Resistant Prostate Cancer	NCT03692663	Recruiting	Metastatic Castration-resistant Prostate Cancer	TABP EIC Cyclophosphamide fludarabine
63	A Study of CNTY-101 in Participants With CD19-Positive B-Cell Malignancies	NCT05336409	Recruiting	R/R CD19-Positive B-Cell Malignancies Indolent Non-Hodgkin Lymphoma Aggressive Non-Hodgkin Lymphoma	CNTY-101 IL-2 Lymphodepleting Chemotherapy
64	Study for Subjects With Relapsed/Refractory Non- Hodgkin Lymphoma	NCT05618925	Not yet recruiting	Non Hodgkin’s Lymphoma Refractory/Relapsed	N803 CD19 t-haNK suspension Cyclophosphamide

#### Low persistence

A major drawback is the lack of in vivo persistence of infused cells in the absence of cytokine support, limiting the effectiveness of NK cell immunotherapy. While exogenous cytokines have been reported to increase proliferation and persistence of adoptive NK cells [139], they can also lead to undesired adverse effects, including the expansion of suppressive immune subsets, such as Tregs [140]. Rejection of allogeneic NK cells by host T cells is also a critical consideration in cell therapy using allogeneic NK cells. Further exploration of the role of transmembrane-bound IL-15 in promoting NK cell persistence is warranted.

#### Transport to the desired tumor site

The efficient homing of NK cells to tumor sites has been debated, as rapid homing to the tumor bed is critical for adoptive cell therapy efficacy. This process is regulated via complex interactions between NK cells and chemokines released by tumor cells [141]. Various engineering approaches have been explored to enhance NK cell migration to tumor sites. For instance, NK cells have been subjected to electroporation with mRNA encoding the chemokine receptor CCR7 to enhance migration to lymph nodes expressing the chemokine CCL19 [20, 142]. To improve the success rate of NK cell immunotherapy in patients with solid tumors, mouse models have been used to explore novel techniques promoting NK cell translocation to tumor sites [143]; however, the effectiveness of these approaches requires further verification in clinical trials.

#### Immunosuppressive tumor microenvironment (TME)

The TME, encompassing immunosuppressive molecules, immunosuppressive cells, and an unfavorable environment hindering immune cell function, poses a major obstacle for CAR-NK cell therapy. Immunoregulatory factors such as transforming growth factor (TGF)-β and others present in the TME can impair NK cell activity [144]. Researchers are investigating the development of CAR-NK cells that counteract some of these immunosuppressive effects, such as knocking out associated genes of NK cells using CRISPR/Cas9 technology [145]. Another strategy to overcome NK cell depletion [146] in the TME is to eliminate checkpoint components using genome editing to improve their function.

#### Low transduction efficiency of lentivirus

Lentivirus-based transduction systems represent one of the most commonly used methods for intracellular gene modification and delivery. However, the natural resistance of NK cells to lentivirus poses a challenge to efficient transduction. Various chemicals, such as protamine sulfate, are employed to enhance viral transduction [147].

Altogether, while CAR-T cell immunotherapy provides a promising approach to treating certain cancers, there are still several limitations: 1) high costs leading to unavailability; 2) long production cycles resulting in patients being unable to afford waiting; 3) poor cell quality of patient samples potentially leading to production failure; 4) CRS and neurotoxicity contributing to high treatment risks. Therefore, the general CAR holds great promise. NK cells, with their unique biological characteristics, demonstrate distinct advantages as potential “off-the-shelf” universal CAR-NK cells. As a promising alternative, different sources of NK cells (including UCB, PB, cellular lines, and iPSCs) could be utilized. Additionally, the allogeneic context without obvious toxic adverse effects presents a significant advantage, even though most CAR-NK cells are still in preclinical or early clinical trial stages. However, the short persistence of NK cells after infusion in vivo remains a major setback. Optimization and standardization of cell expansion and target gene transfection also need further definition, considering the differences between T cells and NK cells. Lastly, akin to CAR-T cells, the lack of tumor-specific targets poses a significant challenge for CAR-NK cell applications in various cancer treatments, including hematologic and solid tumors, necessitating further development for precision medicine.

## Development directions of NK cell therapy

### Recognition of novel target antigens

As mentioned above, identifying highly consistently expressed target tumor antigens is a critical step in CAR design. Most TAAs are also expressed by some healthy cells, potentially causing a “targeting nontumor” effect [148]. Furthermore, the expression of these TAAs can vary greatly among single-cell clones of the same tumor. To address this issue, bispecific CARs have been designed to target multiple antigens simultaneously. This can be achieved by injecting different CAR-NK cells targeting distinct antigens simultaneously or designing one CAR to recognize multiple antigens through “tandem CARs,” wherein two combined elements are attached to individual molecules to enhance the immune synapse [149]. Additionally, multiple CARs can be simultaneously produced on the same immune cell using a vector.

### Improving NK cell activity

Various immune checkpoints, such as PD-L1, regulate and suppress NK cell activity. For instance, a new NK-92 cell line designed with a CAR targeting PD-L1, known as PD-L1-targeting haNK, demonstrated specific antitumor effects against several tumors in preclinical data [150]. Another strategy to improve the activity of CAR-NK cells involves regulating tumor metabolism, an area that has not received sufficient attention. Under hypoxic conditions, adenosine is produced via ATP metabolism by CD39 and CD73, contributing to immune evasion, preventing NK cell trafficking to tumor sites, and inhibiting NK cell maturation. NKG2D-engineered CAR-NK cells exhibited efficacy in treating lung cancer following anti-CD73 antibody inhibition [151]. Therefore, immune checkpoint regulation remains a crucial consideration in cell-based immunotherapy.

### Overcoming the immunosuppressive TME

Tumors harbor various immunosuppressive factors, including TGF-β, IL-10, and PD-1. Several strategies are utilized to mitigate their inhibitory effects. Combining TGF-β kinase inhibitors with NK cells has been observed to restore NK cell cytotoxicity and preserve NKG2D and CD16 expression [152]. Additionally, hybrid CARs incorporating extracellular TGF-β receptor domains have proven successful in improving the antitumor potential of NK-92 cells [153]. Furthermore, the concurrent use of immune checkpoint blockade inhibitors presents a promising avenue.

### Improving security

Enhancing the safety of CAR-NK cell-based therapy may involve modifying the CAR structure by incorporating suicide genes [154]. Developing bispecific CAR molecules to better target tumor-specific antigens is another crucial approach. CAR-NK cells exhibit the unique ability to target tumors in both a CAR-dependent and CAR-independent manner. This ability can be harnessed to achieve enhanced tumor inhibition by developing nonsignaling CARs. These nonsignaling CARs lack direct killing signals but can augment the specific killing of NK cells by promoting residence and adhesion to target cells [136]. Another intriguing strategy involves designing CAR-NKs capable of modulating the TME. These highly specialized CAR-NK cells express several foreign genes that can modulate the local TME to prevent any harmful effects.

### Improving accessibility

Addressing the accessibility of CAR-NK cells in solid tumors necessitates various approaches, including topical, intraperitoneal, and focused ultrasound-guided drug delivery. For instance, pleural injections proved highly effective in an orthotopic model mimicking human pleural malignancies, demonstrating an even longer duration of function compared to intravenous injections [155]. The topical administration of CAR immune cells may also help reduce treatment doses.

## Prospective future

**Fig. 8:**
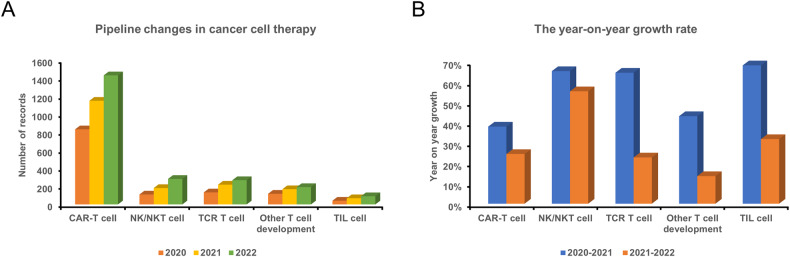
Updated pipelines in cancer cell therapy. Clinical pipeline changes and the year-on-year growth rate of cell therapy in the past 3 years. NK natural killer, TCR T cell receptor, TIL tumor-infiltrating lymphocyte.

NK cells stand as a unique cohort of antitumor effector cells, wielding functions such as MHC-independent cytotoxicity, cytokine production, and immune memory. These attributes position them as pivotal contributors to both innate and adaptive immune response systems. The field of CAR-NK cell therapy holds promise in clinical research, demonstrating commendable safety and preliminary efficacy in certain patients with cancer. In comparison to CAR-T cells, CAR-NK cells boast distinct advantages, yet they grapple with challenges. Enhancing cell proliferation, facilitating more efficient activation of cytotoxicity, and ultimately optimizing NK cell reconstitution are concerns. Consequently, advancements in large-scale preparation methods, cryopreservation measures, and efficacy are imperative. Addressing the short duration of in vivo persistence and exhaustion remains an unresolved frontier. Overall, CAR-NK is poised to evolve into a versatile cell product, holding greater advantages in single-drug or combined transplantation, monoclonal antibody applications, and other treatments. With the formidable antitumor lineage of NK cells as a foundation, overcoming these challenges is likely to usher in groundbreaking developments in tumor treatment. The rapid evolution of NK cell-based immunotherapy (Fig. 8), reflected in the expanding cancer cell therapy pipelines [156, 157], proves that CAR-NK modifications will pave the way for new breakthroughs. In the near future, the maturation of CAR-NK cell therapy technology promises uplifting news for a broader spectrum of patients with cancer, propelling humanity closer to conquering the challenges of refractory and recurrent cancer treatments.

## Data Availability

The relevant information is available from the corresponding author upon reasonable request.
